# The Necessity for Accurate Pre-Operative Diagnosis and the Methods to Be Employed in Intra-Abdominal Lesions

**Published:** 1913-07

**Authors:** Herman E. Hayd

**Affiliations:** 493 Delaware Ave., Buffalo, N. Y.


### BUFFALO MEDICAL JOURNAL

VOLUME 68 JULY, 1913 No. 12

### ORIGINAL ARTICLES

*No responsibility is assumed by the Journal for the opinions of contributors of original articles; nor for methods of expression nor errors in proof reading, excepting for articles in a foreign language contributed for translation. The right is reserved to decline contributions on account of bona fide lack of space; or because they do not bear on practical medicine and surgery; or because they would fail to interest or would give offense to the majority of subscribers. The expression of minority views or reasonable and courteous criticism of the views of others is not considered offensive.*

### The Necessity For Accurate Pre-Operative Diagnosis and the Methods to Be Employed in Intra-Abdominal Lesions

HERMAN E. HAYD, M D., M.R.C S. (Eng.)
Buffalo

ACCURATE diagnosis for the proper treatment of any disease is of the greatest importance, and especially so when we are considering lesions which involve the abdominal organs, where operative undertakings, no matter how trivial, are often matters of the gravest responsibility, not only because life is in jeopardy, but because of the resulting adhesions, which are often consequent upon the opening of the peritoneal cavity. Again, if certain acute fulminating inflammations are not quickly and properly interpreted, a few hours' delay might be the cause of a fatal termination, no matter how carefully or how judiciously the necessary operation was conducted, or certain complications might result from delay in properly diagnosing the case so as to render a simple and comparatively safe surgical undertaking exceedingly dangerous and hazardous. There is practically no mortality incident to modern surgery when skillfully and scientifically executed, but post-operative adhesions, with all their painful and dangerous complications and consequences, their associated distress, future suffering and disability, make the conscientious surgeon hesitate in recommending operative procedures, unless a recognizable pathology of serious possibilities is present, or symptoms which render life miserable or seriously disturb the functions of one or more organs, either, because they are directly involved, or by reason of intimate reflex association.

I shall not attempt to discuss in this paper tonight the symptomatology of the many lesions which can exist in the abdominal cavity, nor of the treatment and surgery which such diseased conditions call for, but only in a very general way develop a few of the important points which must be relied upon to establish a correct diagnosis, and incidentally to bring out a few of the strong and salient features which, when considered, are of inestimable value in building up a clinical picture, and, above all, to impress upon you, that in my experience time and patient study are the two requisites necessary to get this information. Errors in diagnosis are often made, but most frequently, not from want of knowledge on the part of the attending surgeon, but from carelessness and haste in arriving at conclusions.

First of all, a careful history must be taken, and it is easiest and best to listen patiently to all the person wishes to divest himself of, and then proceed to a physical examination, not only of the organ, but organs, which are in the main responsible for the symptoms the patient suffers from, as well as the body in general. The examination is usually best made with the patient lying on the back, and the examiner's hands should be warm and the sufferer should be made as comfortable as possible. First, the upper abdomen should be carefully palpated, and any points of undue prominence must be noted and areas of pain or tenderness or resistance or fluctuation. The hand should be pressed deeply into the right and left flanks, and the kidney carefully palpated. The edge of the liver should be felt for, and its area percussed for lessened or increased dullness. The stomach should be outlined, when such a course is thought necessary, by giving the patient a teaspoonful of bicarbonate of soda in a glass of water, and follow this immediately by a teaspoonful of tartaric acid dissolved in a like amount of water, or the organ may be distended by air from a Davidson syringe. The colon and vermiform appendix are then palpated, and the sigmoid flexure and colon examined for faecal accumulations and growths, and then a careful vaginal examination should be made by touch and inspection. The color of the vagina should be observed, and the presence of discharge, or other evidences of irritation about the labia or clitoris should be looked for. Changes in the position of the uterus and peculiarities of form, size and consistence of the organ should be studied, and alterations in sensibility recognized, and inflammatory masses or adventitious growths felt for—Kelly. The pelvic organs and rectum should be examined by the index finger or two fingers, to note every possible deviation from health, and supplemented by a careful bi-manual examination. Often the patient should be placed in the knee-elbow position, and with instruments of precision the bladder and rectum should be carefully inspected under electric illumination, or by the head glass with reflected light; in fact, everything known to modern medicine and surgery should be employed to get as perfect a clinical picture as is possible. Pain is, perhaps, the most important symptom which causes a sufferer to seek relief, and it may be the result of some existing inflammatory condition in one of the abdominal viscera, or it may be due to some peripheral irritation, or be dependent upon some systemic infection or constitutional dyscrasia as gout, lithaemia, malaria, plumbism and certain anaemias. Dyspeptic phenomena, loss of appetite, distress after eating, pain and even excruciating agony, such as is seen in lead colic, bowel disturbances, loss of general nutrition and strength are symptoms common to many intra-abdominal diseases, and they may also be simply expressions of some irritative condition altogether outside of the peritoneal cavity, as the gastric crises of locomotor ataxia, and thus their causes must be carefully studied lest grave mistakes result from various operative undertakings, which would, perhaps, be justifiable where the symptoms clearly dependent upon an intra-abdominal lesion.

Acute conditions, such as gastric and duodenal perforations, acute gangrene with perforation of the gall bladder, acute gangrenous appendicitis, acute pancreatitis, acute thrombosis of the mesentery, typhoid perforation, and extra-uterine pregnancy, in fact, rupture of any of the viscera, are among the catastrophies which call for immediate and heroic surgery, and mistakes in diagnosis will of necessity be frequent, because such conditions cannot be leisurely studied, nor can time be given in order to obtain the valuable assistance which comes from a careful physical examination of the patient, as well as the added information which can be gotten from thoroughly scientific chemical and laboratory findings, and then, above all, the patient is so desperately ill and so full of pain and suffering that a satisfactory physical examination is impossible. It is, however, in this cataclysmic class of cases where the history of the attack is of the greatest importance, and upon it a diagnosis can often be made, and the great triumphs of modern surgery accomplished. In the elucidation and differentiation of the more common intra-abdominal maladies, especially where there is a history of chronicity, the value of the anamnesis cannot be over-estimated, and perhaps nowhere is this more strikingly illustrated than in gastric and duodenal ulcer, cholelithiasis and appendicitis, diseases which have so many common symptoms and yet certain characteristic signs, which are peculiarly their own. In all, there is a history of stomach distress, perhaps pain, of previous dyspepsia, fullness and weight after eating, eructations of gas, belching and sour stomach, loss of weight and general physical strength.

Pain is the significant landmark, and with ulcer, peptic or duodenal, it is peculiar in that it comes on after eating in from two to five hours, according to the situation of the ulcer. It is circumscribed to the region of the navel. At first it may be complained of only after a hearty meal, or after one meal, and then it becomes evident after each meal. It may persist for days, or even months and years, and then comes a period of quiescence; in fact, this cycle of alternating attack and interval of relief is peculiarly and forcibly the history of ulcer, and remains so unless the ulcer is permanently healed or makes for itself adhesions to other structures, when a symptom complex is added, due to the new involvement and perhaps malignancy. The pain of cholecystitis differs from ulcer in that it is sudden and severe. It has a wider field of radiation. It comes with no regularity as to time. It is rarely caused by food, and it is rarely eased by it. Nor does the patient trace his distress to it. The pain is not gnawing, boring and burning in character, as in ulcer, nor is it relieved by food, hot drinks or alkalines, as in ulcer; nor by vomiting or irrigation which relieves the acid-offending materials of ulcer—Graham. The direction of the pain is a matter of great diagnostic importance and value. The pain of ulcer of the stomach does not radiate. It is central and in the pit of the stomach, while that of gallstones extends over a larger surface and radiates even to the right or left shoulder blade. The pain of nephritic stone runs down the sides following the course of the ureter into the testicle and head of the penis, while that of appendicitis, which although at first is epigastric, soon locates itself in the lower right quadrant and then often extends down the anterior part of the leg, following the course of the anterior crural nerve and its branches. Then, too, the manner in which the subsequent symptoms follow the initial pain is so clear-cut and incisive that a diagnosis can be made with almost unerring certainty. For example, in acute appendicitis, pain is first, often acute and lancinating, then nausea and vomiting, local sensitiveness and spasm of muscle, elevation of temperature and an increase in the number of leukocytes—and this is the uniform order of their appearance. These facts are especially important in differentiating acute appendicitis from lobar pneumonia and diaphragmatic pleurisy, where pain follows the fever and other symptoms of constitutional disturbance. However, in children mistakes in diagnosis are not uncommon, and all of us have perhaps more than once opened the belly in a child and found the appendix normal, and on the following day found the symptoms and signs of a well-marked pneumonia. Of course, the value of the anamnesis depends to some extent upon the degree of intelligence of the patient, who is being investigated, but I am sure more often upon the patience and tact of the man who is doing the investigating. Leading questions must not be asked, and children and nervous persons must be quietly and kindly dealt with, and it is often surprising to see what valuable information they give and here and there suggest a sign or symptom which the astute surgeon can follow up and elaborate, and thus complete a diagnosis. Unfortunately, all of the inflammatory diseases of the upper right quadrant become complicated by associated adhesions and extensions, and then a differential diagnosis often can only be made by an exploratory laparotomy, and then the surety of relief must be left to comprehensive surgery.

Next in importance to careful history-making is the physical examination of the patient. Nature in her effort to protect herself produces spasm of muscle, and, therefore, all inflammatory lesions have pain upon pressure and fixity and rigidity of muscle over the part involved. This pain and tenderness may be very acute, and especially so where the trouble is not too deep-seated. Sometimes, owing to great nervous susceptibility and inability of the patient to relax, an anesthetic must be administered or a hot bath to make the examination sufficiently complete and thorough, and this is especially so for certain pelvic conditions where long, fragile adhesions are present which permit considerable motility and movability of the organs, but yet excursions not great enough to enable them to normally perform their functions, or adhesions and contractures of tissue and structures may exist which are not gross enough to palpate through a tense and rigid vaginal vault. Because the difficulties in diagnosis in abdominal diseases are so great—in fact, sometimes impossible—every resource of value must be at hand to assist us in solving these complex problems. Dr. Robert T. Morris of New York has given us some valuable suggestions in a paper published in the January 25, 1908, number of the Journal of the American Medical Association, and when carefully studied and applied often help to clear up an otherwise obscure case. He has noted that the lumbar ganglia of the sympathetic get sensitive under certain irritative and inflammatory influences, and this tenderness is reflected forwards to a point on each side of the navel. He says: "Draw a line from the right anterior superior spine of the ilium to the navel. On this line, an inch and one-half from the anterior superior spine we find McBurney's point. Tenderness on deep pressure at this point gives presumptive evidence of an irritative process in the vicinity of the vermiform appendix, but tenderness on superficial pressure at this point may mean irritation of sensory nerves of the abdominal wall, and this irritation may be due to causes ranging from hysteria to toxemia. If we come out on this line one and one-half inches from the navel, we get a point of definite diagnostic value, and, according to Morris—in any pelvic disease, whether tubal or ovarian, in old scars in the cervix, metritis, endometritis, piles or bladder inflammations, there will be tenderness over this point on both sides one and one-half inches from the navel.

If the appendix alone is involved, then the tender point is only on the right side. He also says that involuting appendices and those in which the organ is only congested, as from a prolapsed kidney, there will always be seen more or less marked tenderness over this Morris point, while the McBurney point may show no tenderness, even on deep pressure. When the kidneys or gall bladder are affected, there is no point of tenderness over either lumbar ganglia. Sometimes these points of tenderness are matters of great importance in eliminating the causes of many intra-abdominal symptoms when they are due to peripheral irritations, as certain eye reflexes and phorias, which often occasion all kinds of dyspeptic phenomena. Then, too, they are of value when considering the reflex dyspepsias which are seen in so-called uncomplicated retroverted uteri or prolapsed ovaries, or to the painful and tender old scars often noted in healed cervical lacerations.

I have studied these Morris points of pain and tenderness for a number of years, and although I am not prepared to go as far as he does in their constant presence with these pelvic and appendicial lesions, still I have met them often enough to consider their presence as valuable diagnostic aids, and in connection with all the other many signs and symptoms of diseased conditions they simply accentuate the axiom—that to be of value they must be intelligently interpreted—because accurate diagnosis means correct observation and logical deductions.

The Murphy five diagnostic methods for examination of patients are also of great value:

First, the fist percussion of the kidney, which is used to determine the presence or absence of an acute pathological condition within that organ. The patient with clothing removed above the waist is seated in an upright position on a stool, and is then instructed to bend forward as far as possible, and while in this position the examiner's left hand is pressed firmly upon the back, and the right clenched hand is brought down with considerable force upon the dorsum of the left hand, when the patient will cry out with pain if there be an acute infection, infarction or retention in the pelvis of the kidney or stone or ureteral obstruction, because any organ under tension will make pain when suddenly struck.

His second method is used in acute infections of the gall bladder or acute obstruction of the cystic or common duct, with or without infection. The examiner sitting at the right side of the recumbent patient presses his left finger deeply under the costal arch at the tip of the ninth cartilage. The patient is then asked to take a deep inspiration, which forces the gall bladder below the costal guard or border, and when the flexed finger is suddenly struck with the ulnar side of the right hand, great pain is provoked. This he calls his hammer stroke percussion. The third method—which is used also for gall bladder examinations—is called the deep grip palpation, which is simply squeezing the tender gall bladder up against the diaphragm as it descends with each inspiratory effort. A fourth is the piano percussion to demonstrate small quantities of fluid and exudate in the abdominal cavity, and is simply tapping the abdominal wall with the tips of the four fingers, one after the other, commencing with the index finger; and a fifth method—the simultaneous palpation of both iliac fossa for suspected acute appendicial involvement.

Intra peritoneal conditions often manifest themselves in swellings and growths, and these masses may be painful and tender, or without any special sensibility. They may be movable or fixed, and in some cases they wander about the abdominal cavity swinging upon their stretched-out pedicles. They can in most cases be felt by careful palpation, if the patient be placed in various positions which relax superimposed organs or tense muscles. In thin subjects their outline can be carefully and thoroughly studied, but if the person be very fat, often this physical disproportion makes a careful examination impossible. A very important and possible complication may cause some difficulty, as when a displaced organ, as a kidney or uterus or ovary, becomes fixed in its altered position and then can easily be mistaken for some adventitious growth or a neoplasm—benign or malignant—or a hard growth lying over a large blood vessel may have such increased visible pulsation as to be easily mistaken for an aneurism. In dealing with a swelling in the abdominal cavity, the bowels should be thoroughly unloaded, and the bladder should be emptied, because many errors of diagnosis have resulted from this want of care on the part of the examining surgeon. Phantom tumors must be thought of, and their presence can be easily demonstrated by their disappearance under an anaesthetic. Inspection of the abdomen, when done systematically, opens up a vista of possibilities. Peristaltic waves may be seen coming up to a point and increasing with visible intensity and culminating in crises of pain as in volvulus and bowel obstruction. Swellings in certain regions which commenced on one side or were first observed by the patient in the median line help in differentiating uterine and ovarian tumors, and even hydro-nephroses and occasional, although rare, echinococcic cysts of the liver.

Laboratory methods as employed by every capable and scientific surgeon are, of course, of great value, but often their importance is exaggerated, and, in my judgment, should only be relied upon when the other clinical symptoms accord with the findings. Increased leucocytosis and a great disproportion between the cell elements of the blood and high polymorphia percentages, iodinophilia are of immense value in making a quick decision for operation, and especially when we are dealing with appendicitis, a disease which is responsible for over sixty per cent. of the acute inflammations of the abdominal cavity, and one which should be practically without mortality if medical men would look upon it as a grave surgical malady from its incidence. I believe that a great many deaths in this terrible disease can be traced to the delay consequent upon medical men in trying to make the diagnosis of the danger point by blood counts and other physical and clinical symptoms. After years of patient study and a large experience in dealing with this treacherous malady, I am of the opinion that surgery should be at once resorted to in every frank case of acute appendicitis, and that sudden pain in the lower right quadrant, perhaps extending to the pit of the stomach, vomiting, with fever and rigidity of the rectus muscle establishes a diagnosis with such certainty that laboratory examinations of the blood are unnecessary, and that they lead to delay and uncertainty in the only possible treatment, which should be prompt and efficient surgery.

In a more complicated condition, as when an appendicitis becomes engrafted upon a pelvic inflammation, when, for example, the patient is a woman who has been in bed for a week or more with peritonitis from tubal or ovarian involvement, or in a woman who has previously suffered from a pelvic inflammation, with masses which fill both lateral vaginal vaults, a blood count is of the greatest importance, and gives the surgeon a great sense of comfort and security. Here a sudden accession of pain in the right side, with increased leucocytosis and a high percentage of polymorphia muclear cells, makes an operation imperative, because the pathological condition has changed and there has been added an acute appendicitis, perhaps by extension, and with it the dangers of rupture and a rapidly spreading fatal peritonitis, a complication which is not imminent in the course of simple tubal and ovarian pathology.

Appendicitis should always be operated early, because one cannot tell which attack or which person will develop pus, and so strongly does Murphy feel on this question, that I shall quote him from his August, 1912, Surgical Clinics. He says "that every case of appendicitis that is operated in which pus is found outside of the appendix at the time of operation has, in the presence of that pus, irrefutable evidence that the case was badly managed up to that time."

Laboratory examinations and chemical tests of the gastric contents are also of some value, but I am satisfied that many cases of ulcer of the stomach and duodenum have gone into an unoperable stage by delay, in trusting too much to simple chemistry, and that cancer—over fifty per cent—Mayo—so frequently engrafted upon old ulcer areas, has progressed beyond curative operative limits by diet, tentative and medical treatment, and often under the conservative counsels of the distinguished gastrologists. An absence of free HCl and an increase in the amount of lactic acid and the presence of the Boaz baccilli is only presumptive evidence of cancer, and so an increase in free HCl and high combined acids may exist, and yet the case be one of old ulcer, and upon a certain part of it cancer has been engrafted. Therefore, these useful and scientific methods of examination must carry with them the weight of conviction only when the clinical picture coincides with their possible sequences.

Miscroscopical examinations of the urine are of the greatest importance, and in a certain class of cases they establish the diagnosis. In ureteral inflammations and pyelitis, clumping of pus cells and the recognition of epethelial shedding from the pelvis of the kidney often clears up the cause of certain obscure septic symptoms. Cystoscopic examinations of the bladder and ureteral catheterization are also of great value, and particularly if we believe, as Kelly does, that over sixty per cent. of those cases of ill-defined right-sided pain are due to some trouble in the kidney, and usually a displacement, with a kinking of the ureter and retention of urine in the renal pelvis. Backache, which is also a common symptom in women, Kelly says, is more often due to anaemia and neurasthenia than to uterine and pelvic diseases. However, a retroverted uterus, with its associated endometritis, or a large and tender tube or ovary, varicosities of the broad ligament, or a tender, deflected cocyx, or a painful fissure of the rectum, or ulcer or piles must be looked upon as very common causes of backache and prolonged ill health in women; and varicocele, painful, tender and hyper-aesthetic areas in the male urether, strictures of large calibre often cause very distressing backache in men.

The presence of albumen and casts in surgical cases must not be treated too lightly, nor should their presence be taken too seriously, and especially so when the spigmomanometer shows no increased blood pressure. High blood pressure readings, with the discharge of large amounts of urine, or even with diminished secretion of urine, with perhaps only a trace of albumen, are of infinitely greater importance in establishing the surgical risk of any operative case.

Blood in the stools is of great value. In making a diagnosis in obscure abdominal conditions the presence of macroscopic or microscopic blood may be confirmatory in a suspected condition of hemorrhage into the gastro-intestinal tract. The history of tarry stools, accompanied by symptoms of gastric or duodenal disturbance, may be enough to establish a diagnosis of a gastric or duodenal ulcer. A passage of bright blood would point to ulceration lower down. The chemical test for blood in the stools, the so-called "occult blood" would have no value unless proper precautions were taken to place the patient upon a diet devoid of blood pigments. Blood upon the outside of perfectly formed stools comes from the rectum or anus. If evenly distributed through the feces and of a brown or black color, the blood undoubtedly comes from the small intestines. However, when weighing the evidence of the examination of blood in the feces, considerable care must be exercised in excluding blood in the gastro-intestinal tract from other sources, as the mouth, nose, throat and lungs. And equal care must also be taken in the prevention of any admixture of blood from the vagina during a normal defecation.

The X-ray is also a valuable means in diagnosing intra-abdominal lesions. In the control of fractures and dislocations the ray has attained its highest degree of perfection, and certainly no surgeon to-day thinks of caring for a fracture or dislocation without its aid, and it is a question whether he would not lay himself open to a charge of negligence if he essayed to practice surgery without it. In urological conditions it vies with the cystoscope in brilliantly illuminating the kidney, ureter and bladder, so that stones, malignant growths and even ureteral kinks can be detected. We can also examine the soft structures with precision and with carefully applied Bismuth tests. Visceral ptoses and intestinal twists can be mapped out; pyloric growths, stenosis and adhesions can often be satisfactorily delineated, and by its scientific use and correct interpretation, hysteria and functional diseases and other causes of ill-health and suffering have often been eliminated because of a definite and easily seen pathology present to account for the symptoms. However, one must not forget that even the X-ray has its limitations, and that grave errors can be made in demanding from the radiologist what the skiagraph, the floroscope and even the photograph cannot reveal, owing to the transparent and translucent character of the products of irritation. Indeed, certain kinds of gall stones, and even nephritic stones, are not dense enough to show a shadow, and shadows are often made by other conditions which even the skilled radiologist cannot explain, and thus great care and often much experience is required in properly interpreting even very excellent plates, and many must be made from different angles to get at different conditions.

The various tuberculin tests and reactions must be made, as well as the Wasserman, and above all these tests and valuable aids in diagnosis must be considered with common sense and judgment.

Many surgical errors are committed every day by well-qualified and scientific men who have a superabundance of theory and laboratory knowledge, but they lack the practical application of a few good surgical principles—in fact, they need a little horse sense. Much worse, however, is the position of some, so-called surgeons, ignorant, selfish and unscrupulous men, whose lack of knowledge and shameful paucity of every qualification which is necessary to equip one to be a surgeon and a diagnostician to properly discharge the serious responsibilities of his calling. He may be able to cut well, and he may know the technique of aseptic surgery so that his mortality, even in grave surgical undertakings, may be surprisingly low, yet I believe that I am voicing the sentiments of all of you when I say that it is he who is responsible for most of the unnecessary and mutilating operations which a long-suffering and abused public is justly and righteously rebuking today.

493 Delaware Ave., Buffalo, N. Y.

---

Absorption of Fat-like Substances Other Than Fats. W. R. Bloor of St. Louis, *Interstate Med. Jour.*, March, 1913, mixed vaseline, liquid petrolatum and lanolin with absorbable fats and found that 90% or more could be recovered in the feces, and that, in other experiments, none was found in the chyle. He therefore corroborates the prevalent opinion that they are not absorbed and have no value as nutrients.

